# Potential role of *APOE* ɛ4 allele as a modifier for the association of *BDNF* Val66Met polymorphisms and cognitive impairment in community-dwelling older adults

**DOI:** 10.3389/fnagi.2024.1330193

**Published:** 2024-02-05

**Authors:** Shaozhen Ji, Jia Kang, Chao Han, Xitong Xu, Meijie Chen, Jie Chen, Jagadish K Chhetri, Jing Pan, Piu Chan

**Affiliations:** ^1^Department of Neurology, Dongfang Hospital, Beijing University of Chinese Medicine, Beijing, China; ^2^Department of Neurology, The Second Affiliated Hospital of Inner Mongolia Medical University, Hohhot, China; ^3^National Clinical Research Center for Geriatric Diseases, Xuanwu Hospital of Capital Medical University, Beijing, China; ^4^Department of Neurology, Shandong Provincial Hospital Affiliated to Shandong First Medical University, Jinan, China; ^5^Department of Geriatrics, Shenzhen Hospital, Peking University, Shenzhen, China; ^6^Department of Neurobiology, Neurology and Geriatrics, Xuanwu Hospital of Capital Medical University, Beijing, China

**Keywords:** APOE ε4, BDNF Val66Met, cognitive impairment, older adults, modify

## Abstract

**Objective:**

To determine whether the brain-derived neurotrophic factor (*BDNF*) Val66Met polymorphism is associated with cognitive impairment (CI) in community-dwelling Chinese older adults, and to investigate whether this relationship is modified by the Apolipoprotein E (*APOE*) ɛ4 allele.

**Methods:**

The study is a secondary analysis of 703 participants aged ≥60 years randomly enrolled from the Beijing Longitudinal Study of Aging II prospective cohort. The education-adjusted Mini-Mental State Examination and the Clinical Dementia Rating Scale were used to measure the cognitive performance of the subjects. The main effects and interactions (additive and multiplicative) of the *BDNF* Met and the *APOE* ε4 alleles on CI were estimated by logistic regression models.

**Results:**

In total, 84 out of 703 older adults aged ≥60 years old had CI. No significant difference was observed in the risk of CI between participants with the *BDNF* Met allele and that of subjects without the *BDNF* Met allele (*p* = 0.213; *p* = 0.164). Individuals carrying both the *BDNF* Met and *APOE* ε4 alleles had an almost 1.5-fold increased odds of CI compared with carriers of the *BDNF* Met allele but without the *APOE* ε4 allele. The additive association indicated a positive interaction of both *BDNF* Met and *APOE* ε4 alleles with wide CIs (*p* = 0.021; *p* = 0.018).

**Conclusion:**

The results suggest that the *APOE* ε4 allele may be a potential modifier for the association of the *BDNF* Val66Met polymorphism with CI in community-dwelling older adults.

## Introduction

Aging is usually described as a multifaceted deterioration within cognitive disorders and other physical dysfunctions that are known to induce the loss of functional capacity and decreased quality of life in older adults. Aging is often accompanied by a decline in some cognitive domains including memory, learning, concentration, execution, and calculation. However, cognitive dysfunction ascribed to senescence is not equally observed in adults, and a substantial proportion of individuals still maintain healthy cognitive function even into old age. It has been reported that some genetic predisposition to different cellular and molecular neurobiological factors affecting long-term cognitive ability may explain the heterogeneity seen in cognitive performance in older people (Alzheimer's, 2012). Comprehending the genetic sources of heterogeneity in cognitive aging could provide crucial contributions to future endeavors aimed at screening, treatment, or prevention of neurodegenerative diseases such as Alzheimer’s disease (AD).

It has been reported that some cognitive disorders observed in the elderly may be associated with disruptions in the neurotrophic systems (Mattson et al., 2004). Genetic studies have indicated that variations of alleles within the neuroplasticity-related gene encoding for neurotrophins may be potential sources of some individual variations in cognitive aging (Barha et al., 2019). Brain-derived neurotrophic factor (*BDNF*) is characterized to partake in neuronal growth and differentiation and synaptic plasticity, which performs an essential role in memory storage and learning (Song et al., 2015). The human *BDNF* gene located on chromosome 11p14.1, is composed of 11 exons and 9 functional promoters. A guanine to adenine single nucleotide polymorphism (SNP) within the pro-domain region of the *BDNF* gene at position 196 of exon 2 results in an amino acid substitution from valine (Val) to methionine (Met) at codon 66, which is associated with reduced levels of the activity-dependent neuronal secretion of the mature form of *BDNF* (Egan et al., 2003; Chen et al., 2008). Although numerous studies have shown a relationship between Val66Met polymorphism in the *BDNF* gene and cognitive performance, some have failed to establish such an association (He et al., 2007; Bicalho et al., 2018; Zhao et al., 2018); the influence of the Met allele, in particular, has not been determined (Toh et al., 2018). Even the relationship between *BDNF* Val66Met polymorphisms and cognitive function remains controversial (Brown et al., 2020). Previous studies have confirmed that older adults with the Met allele have a higher risk of cognitive impairment (CI) compared to Val homozygotes carriers (de Azeredo et al., 2017; Lim et al., 2018; Tan et al., 2018), and some have found that the Val allele may increase this risk (Ventriglia et al., 2002; Matsushita et al., 2005; Rezaei et al., 2017). These contradictory findings may be partly due to the omission of some confounding factors such as age (Brown et al., 2020), sex (Li et al., 2017; Barha et al., 2019) and other neurodegenerative pathologies.

The apolipoprotein E epsilon 4 (*APOE* ε4) allele is a major genetic risk factor for sporadic AD (Reiman et al., 2007). In recent years, *BDNF* Val66Met polymorphism has been reported to interact with *APOE* ɛ4 on working memory, verbal and visual episodic memory, and the progression of mild cognitive impairment (MCI) (Forlenza et al., 2010; Ward et al., 2014; Lim et al., 2015; Gomar et al., 2016). While some studies did not indicate the influence of *BDNF*/*APOE* gene–gene interaction on cognitive performance, incidents of AD or MCI (Forlenza et al., 2010; Richter-Schmidinger et al., 2011; Zhao et al., 2018). There is limited information and uncertain conclusions regarding the interactive effect of the *BDNF* Val66Met gene polymorphism and the *APOE* ε4 allele on the risk of CI in community-dwelling elderly. The purpose of this study is to investigate whether the *BDNF* Val66Met polymorphism may confer CI risk through the modifying effect of *APOE* ε4, based on data from the Beijing Longitudinal Study of Aging II (BLSA II) prospective cohort.

## Materials and methods

### Study design and participants

This is a secondary analysis of data from the BLSA II prospective cohort project, which randomly enrolled 10,039 community-dwelling residents aged ≥55 years old in Beijing in 2009 (Ji et al., 2020). All eligible residents provided their informed consent. The research ethics committee of Xuanwu Hospital at Capital Medical University provided approval for the protocol of this study.

DNA samples were available for 8,405 subjects of the BLSA II prospective cohort, among which 730 older adults aged ≥60 years were randomly enrolled in the present study and completed both *BDNF* Val66Met and *APOE* genotype testing. For the current analysis, 27 participants without completed cognitive assessments (*n* = 21) or education data (*n* = 6) were excluded.

### Cognitive measures and CI definition

The global cognitive function of the subjects was measured using the Mini-Mental State Examination (MMSE) and the Clinical Dementia Rating Scale (CDR). As described elsewhere (Cui et al., 2011), CI was defined by the CDR score ≤ 1, and the MMSE score ≤ 17 (illiterate) ≤ 20 (primary school) or ≤ 24 (secondary school or above).

### Assessment of covariates

These covariates were selected *a priori* as potential confounders based on the literature (Stuck et al., 1999; Kalaria et al., 2008). A standardized self-administered questionnaire comprising information on demographics, smoking and drinking history, comorbidities (i.e., cerebrovascular disease, hypertension, heart disease, diabetes, cataracts, hearing impairment) and depression was administered in a face-to-face interview by trained physicians and nurses. Smoking or drinking status was categorized as “never” or “past and current.” Depression was assessed by the 15-item Geriatric Depression Scale (GDS) with a score of ≥5 (Almeida and Almeida, 1999).

### Genotyping

DNA was isolated from peripheral venous blood using the standard phenol-chloroform method. Genotyping of *APOE* polymorphism (*APOE* ε2, 3, 4) and *BDNF* Val66Met polymorphism was examined by polymerase chain reaction (PCR) (Xiu et al., 2017). The following primers were designed to amplify *APOE* and *BDNF* genes, respectively: (*APOE*) 5′-TCCAAGGAG-GTGCAGGCGGCGCA-3′ (forward) and 5′-ACAGAATTCGCCCCGGCCTGGTACACTGCCA-3′ (reverse); (*BDNF*) 5′ -GGACTCTGGAGAGCGTGAA-3′ (forward) and 5′ -CGTGTACAAGTCTGCGTCCT-3 (reverse). Genotyping of the PCR products was subsequently performed using Sanger sequencing. *BDNF* Val66Met and *APOE* genotype assays and calls were conducted by researchers blinded to clinical data.

### Statistical analysis

Allele and genotype frequencies were determined by counting and calculating sample proportions, and the Hardy- Weinberg equilibrium was estimated by the Chi-squared (*χ*^2^) test. Continuous variables were described as mean ± standard deviation (SD) and analyzed by the Mann–Whitney U test based on distributional properties. Categorical variables were presented as percentages and frequencies and analyzed by the χ^2^ tests.

Participants were defined as *BDNF* Met allele carriers who had at least one allele of Met (Val/Met and Met/Met), and the others were defined as *BDNF* Met allele (Val/Val) negative. The association of the *BDNF* Val66Met polymorphism with CI was assessed by binary logistic regression analyses, which were performed with initial adjustment for age, sex, and years of education (model 1), and further adjustment for the variables in model 1 plus other potential confounding factors (model 2).

All individuals were divided into four groups to estimate possible joint effects of the *BDNF* Met and the *APOE* ε4 alleles on CI: participants carrying neither the *BDNF* Met allele nor the *APOE* ε4 allele (group A, reference group), participants carrying the *BDNF* Met allele but not the *APOE* ε4 allele (group B), participants without the *BDNF* Met allele but with the *APOE* ε4 allele (group C) and participants with both the *BDNF* Met and *APOE* ε4 alleles (group D). The risk of CI in all groups was tested by binary logistic regression models (model 1 and Model 2). Possible additive effects of the *BDNF* Met and *APOE* ε4 alleles were examined by comparing the odds ratios (ORs) of group D with the summed ORs of groups B and C, with a calculation of the relative excess risk due to interaction (RERI) (RERI score>0: positive additive interaction; RERI score<0: negative interaction) (Hosmer and Lemeshow, 1992; Muller-Gerards et al., 2019).

The effect of the *APOE* ε4 allele on the relationship between the *BDNF* Val66Met polymorphism and CI was calculated with two logistic regression models (model 1 and model 2). The ORs for incident CI in *APOE* ε4 individuals carrying the *BDNF* Met allele were compared with the ORs in the subjects without the *BDNF* Met allele. The measure of interaction between the *BDNF* Met and *APOE* ε4 alleles on a multiplicative scale was calculated based on the following logistic regression model (de Mutsert et al., 2009):

Ln [p/ (1−p)] = β0 + β1⁎ *BDNF* Met + β2⁎ *APOE* ε4 + β3⁎ *BDNF* Met⁎ *APOE* ε4 [p/ (1−p): the odds of the outcome, p/ (1−p) =1: no interaction, p/ (1−p)>1: positive interaction, p/ (1−p) <1: negative interaction; β3: the regression coefficient of the modification effect on a multiplicative scale]

Statistical significance was set at a two-tailed *p* value <0.05. SPSS version 25.0 (IBM Corp, Armonk, NY, United States) and R Statistical Software (version 3.4.2; R Foundation for Statistical Computing, Vienna, Austria) were used for data analysis.

## Results

The full sample of this study consisted of 703 participants which included 84 (11.95%) individuals with CI and 619 subjects with normal cognitive function. There were 210 subjects carrying (29.87%) Val/Val homozygotes, 350 (49.79%) with Val/Met heterozygotes, and 143 with (20.34%) Met/Met homozygotes, with no deviation from the Hardy–Weinberg equilibrium (*p* = 0.99). A total of 264 individuals carried at least one *APOE* ε4 allele. Table 1 shows the demographic and clinical characteristics.

**Table 1:** Study participant characteristics by cognitive function status.

Characteristics *n*%	CI *N* = 84	NC *N* = 619	*p*-value
Age (years), mean (SD)	72.63 (6.87)	69.73 (6.65)	<0.001
Sex: male, *n* (%)	26 (30.95)	245 (39.58)	0.127
Years of education, mean (SD)	7.68 (4.38)	8.25 (4.23)	0.277
MMSE scores, mean (SD)	18.48 (5.39)	27.57 (2.78)	<0.001
Depression, *n* (%)	14 (16.67)	80 (12.92)	0.344
Cerebrovascular disease, *n* (%)	14 (16.67)	71 (11.47)	0.170
Hypertension, *n* (%)	42 (50.00)	334 (53.96)	0.495
Heart disease, *n* (%)	17 (20.24)	130 (21.00)	0.872
Diabetes, *n* (%)	21 (25.00)	135 (21.81)	0.509
COPD, *n* (%)	2 (2.38)	24 (3.88)	0.495
Tumor, *n* (%)	1 (1.19)	15 (2.42)	0.477
Cataract, *n* (%)	14 (16.67)	63 (10.18)	0.074
Hearing problems, *n* (%)	8 (9.52)	28 (4.52)	0.051
Past or current alcohol use, *n* (%)	9 (10.71)	92 (14.86)	0.309
Past or current smoking, *n* (%)	16 (19.05)	131 (21.63)	0.655
*BDNF* genotypes, *n* (%)			0.507
*BDNF* Val/Val	21 (25.00)	189 (30.53)	
*BDNF* Val/Met	43 (51.19)	307 (49.60)	
*BDNF* Met/Met	20 (23.81)	123 (19.87)	
*APOE* ε4 allele, *n* (%)	39 (46.43)	224 (36.19)	0.069
ε4/ε4	4 (4.76)	33 (5.33)	

Results of binary logistic regression analyses for the independent influence of the *APOE* ε4 or *BDNF* Met alleles on CI are shown in Table 2. Participants carrying the *APOE* ε4 allele had a higher risk of CI compared to those without the *APOE* ε4 allele (*p* < 0.001 for model 1 and model 2). There was no significant variation in the distribution of CI status between participants with the *BDNF* Met allele and subjects without the *BDNF* Met allele (*p* = 0.213 for model 1; *p* = 0.164 for model 2).

**Table 2:** Odds ratios for CI by the occurrence of the *APOE* ε4 allele and the *BDNF* Met allele.

	Model 1		Model 2	
	OR (95% CI)	*p*-value	OR (95% CI)	*P*-value
*BDNF* Met allele	1.405 (0.823–2.397)	0.213	1.476 (0.853–2.553)	0.164
*APOE* ε4	2.325 (1.388–3.893)	0.001	2.683 (1.512–4.603)	0.001

Model 1: adjusted for age, sex, education.

Model 2: adjusted for all the comorbidities listed in Table 1.

CI, confidence interval; OR, odds ratio.

Tables 3, 4 show the results of logistic regression analyses for the interactive effect of the *APOE* ε4 and *BDNF* Met alleles on CI, with the highest OR for CI in group D (*p* = 0.006 for model 1; *p* = 0.003 for model 2). Among *APOE* ε4 allele carriers, individuals with the *BDNF* Met allele showed an almost 1.5-fold higher OR for CI compared with subjects without the *BDNF* Met allele (*p* = 0.041 for model 1; *p* = 0.036 for model 2), which was not observed in participants without the *APOE* ε4 allele (right column in Tables 3, 4). RERI scores indicated a positive additive interactive effect of both the BDNF Met allele and the *APOE* ε4 allele on the risk of CI (*p* = 0.021 for model 1; *p* = 0.018 for model 2). A possible multiplicative positive interaction was detected for the two adjusted models, but neither of the two multiplicative scales reached statistical significance (*p* = 0.081 for model 1; *p* = 0.089 for model 2).

**Table 3:** Risk of CI for *BDNF* Met allele and *APOE* ε4 genotype groups (model 1).

	*BDNF* Met allele (−)		*BDNF* Met allele (+)		OR (95% CI); *P* for *BDNF* Met allele within strata of *APOE* ε4
Risk of incident CI	CI/NC	OR (95% CI)	CI/NC	OR (95% CI)	
Model 1					
*APOE* ε4 (−)	14/117	1 (reference)	31/277	0.928 (0.470–1.831)	0.928 (0.470–1.831)
	Group A		Group B	*p* = 0.829	*P* = 0.829
*APOE* ε4 (+)	7/72	1.108 (0.411–2.984)	32/153	2.787 (1.346–5.771)	2.516 (1.036–6.106)
	Group C	*p* = 0.840	Group D	*P* = 0.006	*P* = 0.041

Effect modification: RERI (95% CI) = 1.751 (0.179–3.323), *P* = 0.021.

Multiplicative scale: ratio of ORs (95% CI) = 2.711 (0.885–8.305), *p* = 0.081.

ORs are adjusted for age, sex, education.

**Table 4:** Risk of CI for *BDNF* Met allele and *APOE* ε4 genotype groups (model 2).

	*BDNF* Met allele (−)		*BDNF* Met allele (+)		OR (95% CI); *P* for *BDNF* Met allele within strata of *APOE* ε4
Risk of incident CI	CI/NC	OR (95% CI)	CI/NC	OR (95% CI)	
Model 2					
*APOE* ε4 (−)	14/117	1 (reference)	31/277	0.978 (0.488–1.963)	0.978 (0.488–1.963)
	Group A		Group B	*p* = 0.951	*P* = 0.951
*APOE* ε4 (+)	7/72	1.238 (0.437–3.509)	32/153	3.285 (1.510–7.150)	2.653 (1.067–6.599)
	Group C	*p* = 0.688	Group D	*P* = 0.003	*P* = 0.036

Effect modification: RERI (95% CI) = 2.069 (0.131–4.007), *p* = 0.018.

Multiplicative scale: ratio of ORs (95% CI) = 2.712 (0.859–8.558), *p* = 0.089.

ORs are adjusted for age, sex, education, smoking, alcohol use, depression, and all the comorbidities listed in Table 1.

## Discussion

In the present study, no independent effect of the *BDNF* Val66Met polymorphism on CI was observed in community-dwelling older adults. Our results showed a higher risk of incident CI in individuals with the *BDNF* Met allele compared with Val/Val homozygotes in *APOE* ε4 allele carriers, and a positive interactive effect of carrying the *BDNF* Met allele and the *APOE* ε4 allele on CI, which suggested that *BDNF* Val66Met polymorphism might confer the risk of CI via the interaction of the *APOE* ε4 allele in community-dwelling older adults.

Many studies have attempted to explore the correlation between Val66Met polymorphisms in the *BDNF* gene and CI with ambiguous findings. While some studies indicated that the *BDNF* Met allele was associated with cognitive dysfunction (Ventriglia et al., 2002; Matsushita et al., 2005; Lin et al., 2014), similar to our findings, other works failed to find this significant relationship (Desai et al., 2005; He et al., 2007; Kim et al., 2011). Our results provided a possible explanation that the *BDNF* Met allele might increase the risk of CI among community-dwelling elderly residents, potentially interacting with the *APOE* ε4 allele. A number of possible reasons could explain the modifying effect of the *APOE* ε4 allele.

First, many lines of evidence suggest that *BDNF* Val66Met is a downstream mediator of amyloid beta (Aβ) toxicity on hippocampal function (Lim et al., 2013). Lim et al. found that subjects with the *BDNF* Met allele showed significant cognitive decline as compared to Val/Val homozygotes, in healthy individuals with high levels of Aβ accumulation. *BDNF* Val66Met was not observed to be associated with cognitive deficits in subjects with low Aβ. These findings show that carrying the *BDNF* Met allele could hasten the onset of clinically significant cognitive dysfunction related to the presence of a high brain Aβ load (Lim et al., 2015). Extensive brain Aβ deposition is well known to be associated with *APOE* genotype, particularly with the number of *APOE* ε4 alleles (Verghese et al., 2011; Villemagne et al., 2011; Liu et al., 2013). It has been reported that among *APOE* ε4 carriers, carrying the *BDNF* Met allele correlates to a greater Aβ load compared to Val homozygotes, particularly in the precuneus, orbitofrontal cortex, gyrus rectus, and lateral prefrontal cortex (Adamczuk et al., 2013). Also, *BDNF* Met within *APOE* ε4 carriers had significantly more amyloid deposition in regions typically affected by AD (Stonnington et al., 2020). This supports our findings that *APOE* ε4 may influence the association of *BDNF* Val66Met polymorphism and CI through Aβ deposition.

Second, the interactive effect of *APOE*/*BDNF* on the integrity of brain functional connectivity may provide another explanation for our findings. Recent work showed slightly decreased functional connectivity within the Dorsal Attention Network (DAN) in *BDNF* Met carriers/*APOE* ε3 homozygotes compared to *BDNF* Met/*APOE*ε4 carriers in healthy older adults (Pietzuch et al., 2021).

However, the mechanism underlying the correlation between *APOE*/*BDNF* interactions and CI remains elusive. It’s reported that the Met allele alters the trafficking and packaging of intracellular pro-BDNF, thereby regulating the secretion of mature BDNF (mBDNF) (Chen et al., 2004). Although the *BDNF* gene Val66Met polymorphism does not appear to affect the secretion of pro-BDNF and mBDNF (Mo et al., 2021), *APOE* ε4 blocks the secretion of mature-BDNF (Rainey-Smith et al., 2014; Sen et al., 2017). Pro-BDNF and mBDNF have opposing biological processes (Lu et al., 2005). There may be a mechanistic link between the *BDNF* Val66Met polymorphism and *APOE* isoforms in regulating mature-BDNF secretion and conversion of pro-BDNF to mBDNF.

Although there have been many suggestions that *BDNF* Val66Met polymorphism is involved in cognitive dysfunction (de Azeredo et al., 2017; Lim et al., 2018; Tan et al., 2018), no clear evidence of the association between *BDNF* Val66Met and CI has been explored in previous studies. The individuals in the current study were randomly selected from community-dwelling elderly subjects in the BLSA II prospective cohort. This increases the authenticity and reliability of the data and reduces recruitment bias. Our study adds cross-sectional evidence to a growing body of literature on the correlation of *BDNF* Val66Met polymorphism with CI. Our findings demonstrate the association of *BDNF* Val66Met with CI, and show the necessity of *APOE* ε4 for this relationship. The strength of these results provides more evidence to re-evaluate the effect of the *BDNF* Met and the *APOE* ε4 alleles on cognitive dysfunction in the elderly.

Our research has several limitations. First, the number of participants was limited, although individuals in the current study were randomly selected from the BLSA II cohort. Second, the MMSE and CDR scores with evidence of a marked ceiling effect were used to define CI, and may have missed important levels of CI. Additionally, the physical activity of older adults, which could potentially affect the association of *BDNF* Val66Met polymorphisms with cognitive function was not included in our analysis. Further longitudinal studies are required to explore the association of *BDNF* Val66Met polymorphisms with CI or other forms of cognitive decline of various etiologies to confirm our findings.

## Conclusion

In conclusion, the results demonstrate the role of the *APOE* ε4 allele in modifying the association between *BDNF* Val66Met and CI in community-dwelling older adults. This finding may provide further evidence to evaluate the influence of *BDNF* Val66Met polymorphism and the *APOE* ε4 allele on cognitive dysfunction in the elderly and contribute to the screening, treatment, or prevention of CI in the future.

## Data availability statement

The original contributions presented in the study are included in the article/supplementary material, further inquiries can be directed to the corresponding authors.

## Ethics statement

The studies involving humans were approved by the research ethics committee of Xuanwu Hospital of Capital Medical University. The studies were conducted in accordance with the local legislation and institutional requirements. The participants provided their written informed consent to participate in this study.

## Author contributions

SJ: Writing – original draft, Data curation, Funding acquisition, Writing – review & editing. JK: Methodology, Writing – review & editing. CH: Data curation, Writing – review & editing. XX: Data curation, Writing – review & editing. MC: Data curation, Writing – review & editing. JC: Data curation, Writing – review & editing. JKC: Writing – review & editing. JP: Conceptualization, Funding acquisition, Project administration, Writing – review & editing. PC: Conceptualization, Funding acquisition, Project administration, Writing – review & editing.

## References

[ref1] Adamczuk K. De Weer A. S. Nelissen N. Chen K. Sleegers K. Bettens K.. (2013). Polymorphism of brain derived neurotrophic factor influences beta amyloid load in cognitively intact apolipoprotein E epsilon4 carriers. Neuroimage Clin 2, 512–520. doi: 10.1016/j.nicl.2013.04.001, PMID: 24179803 PMC3777754

[ref2] Almeida O. P. Almeida S. A. (1999). Short versions of the geriatric depression scale: a study of their validity for the diagnosis of a major depressive episode according to ICD-10 and DSM-IV. Int. J. Geriatr. Psychiatry 14, 858–865. doi: 10.1002/(SICI)1099-1166(199910)14:10<858::AID-GPS35>3.0.CO;2-8, PMID: 10521885

[ref3] Alzheimer's A. (2012). Alzheimer's disease facts and figures. Alzheimers Dement. 8, 131–168. doi: 10.1016/j.jalz.2012.02.001 22404854

[ref4] Barha C. K. Liu-Ambrose T. Best J. R. Yaffe K. Rosano C. Health A.. (2019). Sex-dependent effect of the BDNF Val66Met polymorphism on executive functioning and processing speed in older adults: evidence from the health ABC study. Neurobiol. Aging 74, 161–170. doi: 10.1016/j.neurobiolaging.2018.10.021, PMID: 30448615 PMC6358016

[ref04] Bicalho M. A. Ávila R. Cintra M. T. Soares T. Lage A. F. Souza A. L.. (2018). Effect of BDNF val66met and apoe polymorphism on cognition in older adults. Alzheimer’s and Dementia 14:342.

[ref6] Brown D. T. Vickers J. C. Stuart K. E. Cechova K. Ward D. D. (2020). The BDNF Val66Met polymorphism modulates resilience of neurological functioning to brain ageing and dementia: a narrative review. Brain Sci. 10:195. doi: 10.3390/brainsci10040195, PMID: 32218234 PMC7226504

[ref7] Chen Z. Y. Bath K. McEwen B. Hempstead B. Lee F. (2008). Impact of genetic variant BDNF (Val66Met) on brain structure and function. Novartis Found. Symp. 289, 180–188; discussion 188-195. doi: 10.1002/9780470751251.ch14, PMID: 18497103 PMC2735856

[ref8] Chen Z. Y. Patel P. D. Sant G. Meng C. X. Teng K. K. Hempstead B. L.. (2004). Variant brain-derived neurotrophic factor (BDNF) (Met66) alters the intracellular trafficking and activity-dependent secretion of wild-type BDNF in neurosecretory cells and cortical neurons. J. Neurosci. 24, 4401–4411. doi: 10.1523/JNEUROSCI.0348-04.2004, PMID: 15128854 PMC6729450

[ref9] Cui G. H. Yao Y. H. Xu R. F. Tang H. D. Jiang G. X. Wang Y.. (2011). Cognitive impairment using education-based cutoff points for CMMSE scores in elderly Chinese people of agricultural and rural Shanghai China. Acta Neurol. Scand. 124, 361–367. doi: 10.1111/j.1600-0404.2010.01484.x, PMID: 21303351

[ref10] de Azeredo L. A. De Nardi T. Levandowski M. L. Tractenberg S. G. Kommers-Molina J. Wieck A.. (2017). The brain-derived neurotrophic factor (BDNF) gene val66met polymorphism affects memory performance in older adults. Rev. Bras. Psiquiatr. 39, 90–94. doi: 10.1590/1516-4446-2016-1980, PMID: 28099630 PMC7111449

[ref11] de Mutsert R. Jager K. J. Zoccali C. Dekker F. W. (2009). The effect of joint exposures: examining the presence of interaction. Kidney Int. 75, 677–681. doi: 10.1038/ki.2008.645 19190674

[ref12] Desai P. Nebes R. DeKosky S. T. Kamboh M. I. (2005). Investigation of the effect of brain-derived neurotrophic factor (BDNF) polymorphisms on the risk of late-onset Alzheimer's disease (AD) and quantitative measures of AD progression. Neurosci. Lett. 379, 229–234. doi: 10.1016/j.neulet.2005.01.008, PMID: 15843069

[ref13] Egan M. F. Kojima M. Callicott J. H. Goldberg T. E. Kolachana B. S. Bertolino A.. (2003). The BDNF val66met polymorphism affects activity-dependent secretion of BDNF and human memory and hippocampal function. Cell 112, 257–269. doi: 10.1016/S0092-8674(03)00035-7, PMID: 12553913

[ref14] Forlenza O. V. Diniz B. S. Teixeira A. L. Ojopi E. B. Talib L. L. Mendonca V. A.. (2010). Effect of brain-derived neurotrophic factor Val66Met polymorphism and serum levels on the progression of mild cognitive impairment. World J. Biol. Psychiatry 11, 774–780. doi: 10.3109/15622971003797241, PMID: 20491609

[ref15] Gomar J. J. Conejero-Goldberg C. Huey E. D. Davies P. Goldberg T. E. Alzheimer's Disease Neuroimaging I. (2016). Lack of neural compensatory mechanisms of BDNF val66met met carriers and APOE E4 carriers in healthy aging, mild cognitive impairment, and Alzheimer's disease. Neurobiol. Aging 39, 165–173. doi: 10.1016/j.neurobiolaging.2015.12.004, PMID: 26923413 PMC9969539

[ref16] He X. M. Zhang Z. X. Zhang J. W. Zhou Y. T. Tang M. N. Wu C. B.. (2007). Lack of association between the BDNF gene Val66Met polymorphism and Alzheimer disease in a Chinese Han population. Neuropsychobiology 55, 151–155. doi: 10.1159/000106473, PMID: 17657167

[ref17] Hosmer D. W. Lemeshow S. (1992). Confidence interval estimation of interaction. Epidemiology 3, 452–456. doi: 10.1097/00001648-199209000-00012 1391139

[ref18] Ji S. Wang C. Qiao H. Gu Z. Gan-Or Z. Fon E. A.. (2020). Decreased penetrance of Parkinson's Disease in elderly carriers of Glucocerebrosidase gene L444P/R mutations: a community-based 10-year longitudinal study. Mov. Disord. 35, 672–678. doi: 10.1002/mds.27971, PMID: 31912918

[ref19] Kalaria R. N. Maestre G. E. Arizaga R. Friedland R. P. Galasko D. Hall K.. (2008). Alzheimer's disease and vascular dementia in developing countries: prevalence, management, and risk factors. Lancet Neurol. 7, 812–826. doi: 10.1016/S1474-4422(08)70169-8, PMID: 18667359 PMC2860610

[ref20] Kim J.-M. Stewart R. Bae K.-Y. Kim S.-W. Yang S.-J. Kee-Hyung Park K.-H.. (2011). Role of BDNF val66met polymorphism on the association between physical activity and incident dementia. Neurobiol. Aging 32:551. e5-12. doi: 10.1016/j.neurobiolaging.2010.01.018 20172629

[ref21] Li G. D. Bi R. Zhang D. F. Xu M. Luo R. Wang D.. (2017). Female-specific effect of the BDNF gene on Alzheimer's disease. Neurobiol. Aging. 53, 192 e111–192 e119. doi: 10.1016/j.neurobiolaging.2016.12.023 28202203

[ref22] Lim Y. Y. Hassenstab J. Goate A. Fagan A. M. Benzinger T. L. S. Cruchaga C.. (2018). Effect of BDNFVal66Met on disease markers in dominantly inherited Alzheimer's disease. Ann. Neurol. 84, 424–435. doi: 10.1002/ana.25299, PMID: 30014553 PMC6153076

[ref24] Lim Y. Y. Villemagne V. Laws S. Ames D. Pietrzak R. Ellis K.. (2013). Modulation of beta-amyloid-related cognitive decline by brain-derived neurotrophic factor Val66Met polymorphism in preclinical Alzheimer's disease. Alzheimer's Dementia 9, P446–P447.

[ref25] Lim Y. Y. Villemagne V. L. Laws S. M. Pietrzak R. H. Snyder P. J. Ames D.. (2015). APOE and BDNF polymorphisms moderate amyloid beta-related cognitive decline in preclinical Alzheimer's disease. Mol. Psychiatry 20, 1322–1328. doi: 10.1038/mp.2014.123, PMID: 25288138 PMC4759101

[ref26] Lin Y. Cheng S. Xie Z. Zhang D. (2014). Association of rs6265 and rs2030324 polymorphisms in brain-derived neurotrophic factor gene with Alzheimer's disease: a meta-analysis. PLoS One 9:e94961. doi: 10.1371/journal.pone.0094961, PMID: 24733169 PMC3986375

[ref27] Liu C. C. Liu C. C. Kanekiyo T. Xu H. Bu G. (2013). Apolipoprotein E and Alzheimer disease: risk, mechanisms and therapy. Nat. Rev. Neurol. 9, 106–118. doi: 10.1038/nrneurol.2012.263 23296339 PMC3726719

[ref28] Lu B. Pang P. T. Woo N. H. (2005). The yin and yang of neurotrophin action. Nat. Rev. Neurosci. 6, 603–614. doi: 10.1038/nrn1726 16062169

[ref29] Matsushita S. Arai H. Matsui T. Yuzuriha T. Urakami K. Masaki T.. (2005). Brain-derived neurotrophic factor gene polymorphisms and Alzheimer's disease. J. Neural Transm. (Vienna) 112, 703–771. doi: 10.1007/s00702-004-0210-3, PMID: 15375678

[ref30] Mattson M. P. Maudsley S. Martin B. (2004). BDNF and 5-HT: a dynamic duo in age-related neuronal plasticity and neurodegenerative disorders. Trends Neurosci. 27, 589–594. doi: 10.1016/j.tins.2004.08.001, PMID: 15374669

[ref31] Mo M. Fu X. Y. Zhang X. L. Zhang S. C. Zhang H. Q. Wu L.. (2021). Association of plasma pro-brain-derived neurotrophic factor (proBDNF)/mature brain-derived neurotrophic factor (mBDNF) levels with BDNF gene Val66Met polymorphism in alcohol dependence. Med. Sci. Monit. 27:e930421. doi: 10.12659/MSM.930421 34415897 PMC8406813

[ref32] Muller-Gerards D. Weimar C. Abramowski J. Tebrugge S. Jokisch M. Dragano N.. (2019). Subjective cognitive decline, APOE epsilon4, and incident mild cognitive impairment in men and women. Alzheimers Dement 11, 221–230. doi: 10.1016/j.dadm.2019.01.007, PMID: 30891488 PMC6404645

[ref33] Pietzuch M. Bindoff A. Jamadar S. Vickers J. C. (2021). Interactive effects of the APOE and BDNF polymorphisms on functional brain connectivity: the Tasmanian healthy brain project. Sci. Rep. 11:14514. doi: 10.1038/s41598-021-93610-0, PMID: 34267235 PMC8282840

[ref032] Rainey-Smith S. R. Brown B. Gardener S. Peiffer J. Bourgeat P. Laws S. M.. (2014). Apoe genotype-dependent effects of diet and physical activity on cognition and Alzheimer’s-related pathology: Data from the AiBL study of ageing. Alzheimer’s and Dementia 10:166–230.

[ref35] Reiman E. M. Webster J. A. Myers A. J. Hardy J. Dunckley T. Zismann V. L.. (2007). GAB2 alleles modify Alzheimer's risk in APOE epsilon 4 carriers. Neuron 54, 713–720. doi: 10.1016/j.neuron.2007.05.022, PMID: 17553421 PMC2587162

[ref36] Rezaei S. Asgari-Mobarake K. Saberi A. Keshavarz P. Leili E. K. (2017). Brain-derived neurotrophic factor (BDNF) Val66Met polymorphism and post-stroke dementia: a hospital-based study from northern Iran. Neurol. Sci. 37, 935–942. doi: 10.1007/s10072-016-2520-2 27071687

[ref37] Richter-Schmidinger T. Alexopoulos P. Horn M. Maus S. Reichel M. Rhein C.. (2011). Influence of brain-derived neurotrophic-factor and apolipoprotein E genetic variants on hippocampal volume and memory performance in healthy young adults. J. Neural Transm. 118, 249–257. doi: 10.1007/s00702-010-0539-8, PMID: 21190051

[ref38] Sen A. Nelson T. J. Alkon D. L. (2017). ApoE isoforms differentially regulates cleavage and secretion of BDNF. Mol. Brain 10:19. doi: 10.1186/s13041-017-0301-3 28569173 PMC5452344

[ref39] Song J. H. Yu J. T. Tan L. (2015). Brain-derived neurotrophic factor in Alzheimer's Disease: risk, mechanisms, and therapy. Mol. Neurobiol. 52, 1477–1493. doi: 10.1007/s12035-014-8958-4, PMID: 25354497

[ref40] Stonnington C. M. Velgos S. N. Chen Y. Syed S. Huentelman M. Thiyyagura P.. (2020). Interaction between BDNF Val66Met and APOE4 on biomarkers of Alzheimer's Disease and cognitive decline. J. Alzheimers Dis. 78, 721–734. doi: 10.3233/JAD-200132, PMID: 33044176 PMC10416650

[ref41] Stuck A. E. Walthert J. M. Nikolaus T. Bula C. J. Hohmann C. Beck J. C. (1999). Risk factors for functional status decline in community-living elderly people: a systematic literature review. Soc. Sci. Med. 1982, 445–469. doi: 10.1016/s0277-9536(98)00370-0 10075171

[ref42] Tan C. J. Lim S. W. T. Toh Y. L. Ng T. Yeo A. Shwe M.. (2018). Replication and Meta-analysis of the association between BDNF Val66Met polymorphism and cognitive impairment in patients receiving chemotherapy. Mol. Neurobiol. 56, 4741–4750. doi: 10.1007/s12035-018-1410-4 30382534 PMC6647505

[ref43] Toh Y. L. Ng T. Tan M. Tan A. Chan A. (2018). Impact of brain-derived neurotrophic factor genetic polymorphism on cognition: a systematic review. Brain Behav. 8:e01009. doi: 10.1002/brb3.1009 29858545 PMC6043712

[ref44] Ventriglia M. Bocchio Chiavetto L. Benussi L. Binetti G. Zanetti O. Riva M. A.. (2002). Association between the BDNF 196 a/G polymorphism and sporadic Alzheimer's disease. Mol. Psychiatry 7, 136–137. doi: 10.1038/sj.mp.4000952, PMID: 11840305

[ref45] Verghese P. B. Castellano J. M. Holtzman D. M. (2011). Apolipoprotein E in Alzheimer's disease and other neurological disorders. Lancet Neurol. 10, 241–252. doi: 10.1016/S1474-4422(10)70325-2, PMID: 21349439 PMC3132088

[ref46] Villemagne V. L. Pike K. E. Chetelat G. Ellis K. A. Mulligan R. S. Bourgeat P.. (2011). Longitudinal assessment of a beta and Cognition in aging and Alzheimer Disease. Ann. Neurol. 69, 181–192. doi: 10.1002/ana.22248, PMID: 21280088 PMC3045039

[ref47] Ward D. D. Summers M. J. Saunders N. L. Janssen P. Stuart K. E. Vickers J. C. (2014). APOE and BDNF Val66Met polymorphisms combine to influence episodic memory function in older adults. Behav. Brain Res. 271, 309–315. doi: 10.1016/j.bbr.2014.06.022, PMID: 24946073

[ref48] Xiu S. Zheng Z. Guan S. Zhang J. Ma J. Chan P. (2017). Serum uric acid and impaired cognitive function in community-dwelling elderly in Beijing. Neurosci. Lett. 637, 182–187. doi: 10.1016/j.neulet.2016.11.013, PMID: 27890742

[ref49] Zhao Q. Shen Y. Zhao Y. Si L. Jiang S. Qiu Y. (2018). Val66Met polymorphism in BDNF has no sexual and APOE epsilon4 status-based dimorphic effects on susceptibility to Alzheimer's Disease: evidence from an updated Meta-analysis of case-control studies and high-throughput genotyping cohorts. Am. J. Alzheimers Dis. Other Dement. 33, 55–63. doi: 10.1177/1533317517733037, PMID: 28984138 PMC10852485

